# Editorial: Metabolism and Vascular Diseases

**DOI:** 10.3389/fphys.2022.888676

**Published:** 2022-04-25

**Authors:** Kangkang Zhi, Dongze Zhang, Xiaojing Liu, Wang Han-Jun

**Affiliations:** ^1^ Department of Vascular and Endovascular Surgery, Changzheng Hospital, Navy Medical University, Shanghai, China; ^2^ Department of Emergency Medicine, University of Nebraska Medical Center, Omaha, NE, United States; ^3^ Laboratory of Cardiovascular Diseases and Regenerative Medicine Research Center, West China Hospital, Sichuan University, Chengdu, China; ^4^ Department of Anesthesiology, University of Nebraska Medical Center, Omaha, NE, United States

**Keywords:** metabolism, vascular diseases, editoral, basic research, clinical researc

Vascular disease is now the leading cause of death worldwide, with cardiovascular and cerebrovascular diseases being the main contributors (Andersson and Vasan, 2018; Doria and Forgacs, 2019). However, its pathological mechanism is not definite yet. Currently, various kinds of vascular diseases are supposed to be related to metabolic disturbance (Gao et al., 2020; Marini et al., 2020). Generally, specific vascular disease indicates a disorder in the whole circulatory system (Guľašová et al., 2020; Lorenzon Dos Santos et al., 2020). The systemic metabolic disturbance would result in various kinds of pathologic changes in the circulatory system and undermine vascular homeostasis, involve atherosclerosis, vascular remodeling, etc (Huo et al., 2018; Katakami, 2018; Neeland et al., 2019). Meanwhile, cellular metabolism makes a strong bond with the local vascular lesion, like atheromatosis, endothelium injury, etc (Tabas and Bornfeldt, 2020; Wu et al., 2021). However, the metabolic balance would also be affected by local vascular cells’ physiological regulation, vascular development, and hemodynamics (Smith and Ainslie, 2017; Yang et al., 2020). The characterization of metabolic disturbance and related vascular diseases involves many events and complicated connections, which leave quite a lot of gaps in the field to be further studied. This Topic aimed to cover promising and novel research trends in metabolism and related vascular diseases. Several papers which include Basic or Clinical Medical Research and Review from excellent researchers in multiple fields have been collected to date.

As the dominating part of this Topic, papers of basic research that we primarily collected have revealed some interesting relationships between metabolic abnormalities and vascular diseases. The paper by Yu et al. attempted to illustrate the association between plasma metabolic profiles and cerebral collateral circulation in patients with acute ischemic stroke (AIS). They found that the sphingosine-1-phosphate (S1P) level in plasma showed significant positive correlation with good collateral circulation and which might be a potential diagnostic biomarker for predicting collateral circulation status in patients with AIS. The paper by Xue et al. focused on excavating the potential biomarkers in lacrimal diabetic angiopathy and its potential mechanism. Hub genes App, F5, Fgg, and Gas6 related to the regulation of circulation and coagulation were identified. Meanwhile, certain small molecular compounds were considered that might reverse the altered differentially expressed genes. This study might empower the novel potential targets to treat lacrimal angiopathy and other diabetes-related diseases. The paper by Fan et al. found that myricanol could inhibit proliferation and migration of vascular smooth muscle cells (VSMCs) induced by platelet-derived growth factor-BB by suppressing the platelet-derived growth factor receptor-β and NF-kB p65 translocation. Furthermore, myricanol was found suppressing the intimal hyperplasia in mice with carotid stenosis. The paper by da Costa et al. found the events that HFD-fed leading decreased Nrf2 nuclear accumulation, decreased mRNA expression and activity of Nrf2-regulated enzymes (catalase, heme oxygenase-1, peroxiredoxin and thioredoxin) were prevented in castrated mice. The study indicate that testosterone would downregulate Nrf2, leading to oxidative stress and vascular dysfunction in HFD-fed obese mice.

However, papers of clinical research presented in this Topic have significant discoveries as well. The paper by Hu et al. showed that there was no significant correlation between increased serum uric acid levels and the risk of first stroke in the Chinese adults with hypertension. Meanwhile, risk of the first stroke in patients with hyperuricemia less than 60 years old was significantly higher than control. The paper by Yu et al. was based on bioinformatic analysis of metabolomic and proteomic to reveal that the dysregulation of glutamate and glycine metabolism, upregulated glycolysis and fatty acid synthesis in the endothelial progenitor cells that treated with the oscillatory shear stress.

Meanwhile, the Review paper by Ning et al. with creative perspective summarized the potential mechanism underlying metabolic perturbation that type 2 diabetes mellitus (DM) affect the hypertension (HTN), what may be involved in the metabolism of insulin and angiotensin II, sympathetic nervous system as well as the energy reprogramming.
